# The genetic basis of plants’ battle against witchweeds: linking immune responses to distinct resistance mechanisms

**DOI:** 10.1093/jxb/erad305

**Published:** 2023-09-12

**Authors:** Min-Yao Jhu, Dorota Kawa, Siobhán M Brady

**Affiliations:** Crop Science Centre, Department of Plant Sciences, University of Cambridge, Cambridge, UK; Department of Plant Biology and Genome Center, University of California, Davis, CA, USA; Plant Stress Resilience, Department of Biology, Utrecht University, The Netherlands; Plant Environment Signaling, Department of Biology, Utrecht University, The Netherlands; Department of Plant Biology and Genome Center, University of California, Davis, CA, USA

**Keywords:** Cell type-specific defence, cell wall-based resistance, hypersensitive response, inducible defence, lignin, parasitic plants, post-attachment resistance, *Striga*

## Abstract

This article comments on:

Mutinda S, Mobegi FM, Hale B, Dayou O, Ateka E, Wijeratne A, Wicke S, Bellis ES, Runo S. 2023. Resolving intergenotypic *Striga* resistance in sorghum. Journal of Experimental Botany 74, 5294–5306.

**Parasitic plants of the *Striga* species significantly damage cereals in sub-Saharan Africa. Current agricultural practices are insufficient to manage *Striga* infestation, necessitating sustainable approaches that harness natural resistance mechanisms. Mutinda *et al.* (2023) examined how different genotypes of sorghum plants resist *Striga* after it attacks their roots. By comparing transcriptomes, they found that sorghum activates its immune system, and molecular signatures probably associate with distinct resistance mechanisms. This study will inform the development of *Striga*-resistant sorghum varieties to ward off root parasitic plants.**

## *Striga* species are notorious witchweeds

*Striga* species, commonly named witchweeds, parasitize monocot crops, such as sorghum, maize, rice, and millet (David *et al.*, 2022), threatening food security for nearly 300 million people in sub-Saharan Africa (Ejeta, 2007). With a specialized organ called a haustorium, *Striga* forms xylem–xylem connections to ‘steal’ water and nutrients from its host, which drastically hampers crop growth, sometimes leading to complete eradication of crop fields and up to 100% yield losses (Stanley *et al.*, 2021; David *et al.*, 2022).

The *Striga* life cycle depends on host-derived signals: strigolactones trigger *Striga* seed germination (Bouwmeester *et al.*, 2007; Ejeta, 2007; Kountche *et al.*, 2019; Bunsick *et al.*, 2020, 2022, Preprint), while haustorium-inducing factors enable formation of the penetrative structure from the *Striga* radicle (Bandaranayake *et al.*, 2010). Each *Striga* plant produces up to 0.5 million seeds, which can remain viable in the soil for 20 years (Yoneyama *et al.*, 2010; David *et al.*, 2022). The extremely small size of *Striga* seeds allows effective seed dispersal. Their outcrossing nature further maintains high genetic diversity, enabling rapid *Striga* adaptation to the host resistance mechanisms. These genetic characteristics make *Striga* challenging to eradicate.

## Unveiling modes and mechanisms of post-attachment resistance

Resistance to *Striga* can occur before or after *Striga* attaches to the root. The mechanisms of pre-attachment resistance are relatively well understood (Fishman and Shirasu, 2021; Jhu and Sinha, 2022) and we introduce them in Box 1. Distinct modes of post-attachment resistance and defence mechanisms are described in Box 2. In their recent study, Mutinda and collaborators focused on likely molecular mechanisms associated with two post-attachment resistance modes: mechanical barriers and a hypersensitive response (HR).

**Box 1. Host plant pre-attachment resistance to root parasitic plants**

Pre-attachment resistance refers to the strategies employed by host plants to deter or limit the number of germinated seeds to reduce the number of attachments and consequently invasion of parasitic plants (Fishman and Shirasu, 2021; Jhu and Sinha, 2022). Several mechanisms have been identified in host plants to resist root parasitic plants:

**Reducing seed germination rate**

**Fig. 1.**
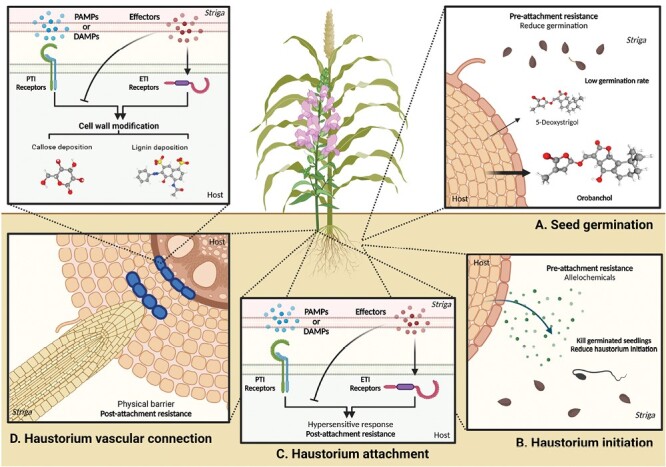
Host resistance responses during different stages of the *Striga* life cycle. (A) Pre-attachment resistance response during *Striga* seed germination. Host plants growing in nutrient-poor soil release strigolactones, promoting beneficial arbuscular mycorrhizal fungus symbiosis. *Striga* seeds perceive these host strigolactones as germination stimulants. However, mutations in genes responsible for strigolactone biosynthesis or alterations in their composition significantly reduce *Striga* seed germination rates. For example, mutations in the *LOW GERMINATION STIMULANT 1* (*LGS1*) gene in resistant sorghum plants alter the composition of strigolactones in root exudates, reducing their stimulatory effect on *Striga* germination. (B) Pre-attachment resistance response during haustorium initiation. Germinated *Striga* seedlings grow towards host roots and perceive haustorium induction factors (HIFs) for haustorium initiation. Resistant host plants produce toxic compounds in root exudates that inhibit the development of parasitic plant seedlings (Box 1). Some resistant host plants produce lower levels of HIFs, reducing *Striga* haustorium formation (Box 1). (C) Post-attachment resistance response during haustorium attachment. Following the detection of pathogen-associated molecular patterns (PAMPs) or damage-associated molecular patterns (DAMPs) from *Striga*, plants initiate pattern-triggered immunity (PTI) to obstruct haustorium attachment. However, parasitic plant effectors can suppress PTI to facilitate parasitism. Consequently, effector-triggered immunity (ETI) overcomes this suppression and triggers hypersensitive responses (HRs) to discourage parasite penetration. (D) Post-attachment resistance response during haustorium vascular connection. Plants fortify cell walls to create physical barriers that hinder the establishment of vascular connections. According to the study by Mutinda *et al.*, cell wall enhancement-based resistance responses probably occur downstream of PTI and ETI. Examples of these barriers include accumulating substances such as lignin or callose in the cortex, impeding the progress of parasites. Moreover, the endodermis serves as a barrier by inducing lignin accumulation, effectively preventing parasitic plant penetration and vascular connection. More details are described in Fig. 2. Three-dimensional structure images of orobanchol (Compound CID: 10665247), 5-deoxystrigol (Compound CID: 15102684), lignin (Compound CID: 175586), and callose (beta-d-glucose, Compound CID: 64689) are exported from PubChem. This figure was created with https://www.biorender.com/.

The seed germination process of *Striga* species is triggered by the presence of strigolactones (SLs), plant hormones commonly found in the root exudates of host plants. However, when mutations occur in genes responsible for SL biosynthesis or alterations in SL composition, germination rates of *Striga* seeds can be significantly reduced (Fig. 1A). One example of this is observed in mutations affecting the *carotenoid cleavage dioxygenase 8* (*ccd8*) gene, which is involved in SL biosynthesis (Gomez-Roldan *et al.*, 2008; Umehara *et al.*, 2008). Such mutations result in a deficiency of SLs, leading to lower germination rates in parasitic plants. Additionally, mutations in the *LOW GERMINATION STIMULANT 1* (*LGS1*) gene in resistant sorghum genotypes cause alterations in the composition of SLs present in root exudates, consequently reducing the stimulatory effect of germination on *Striga* (Fig. 1A) (Gobena *et al.*, 2017).

**Toxic compound secretion**

Some host plants produce toxic compounds in their root exudates that inhibit the germination or development of parasitic plant seedlings (Serghini *et al.*, 2001; Echevarría-Zomeño *et al.*, 2006). For instance, certain resistant sunflower varieties secrete 7-hydroxylated simple coumarins, which create a toxic and hostile environment for the root-parasitic plant *Orobanche* (Serghini *et al.*, 2001). Germinated *Orobanche cernua* seeds near resistant sunflowers exhibit browning symptoms, stunted growth, or even die (Fig. 1B).

**Reducing haustorium initiation**

Haustorium initiation is a crucial step for parasitic plants to establish a connection with the host plant. Multiple studies have highlighted the importance of host-derived haustorium-inducing factors (HIFs) in root-parasitic plants for this initiation process (Kokla and Melnyk, 2018). In response, host plants can exhibit resistance by interfering with the induction of HIFs or disrupting the signalling pathways involved (Rich *et al.*, 2004) (Fig. 1B). Previous studies suggest that certain genotypes of sorghum have a limited ability to initiate the formation of *Striga asiatica* haustoria (Rich *et al.*, 2004). This could also be attributed to the release of substances in the host root exudate that inhibit the induction of haustoria (Keyes *et al.*, 2000).

These findings highlight diverse strategies of host plants to defend against *Striga* and other root parasitic plants. Understanding the mechanisms of pre-attachment resistance opens up possibilities for developing novel approaches to combat *Striga* infestations and improve crop productivity. Further research is necessary to explore the full potential of these mechanisms for their applicability in sustainable agriculture.

**Box 2. Role of the defence mechanisms in post-attachment resistance against parasitic plants**

Natural variation in sorghum cultivar resistance to *Striga* can be used to elucidate target pathways to enhance resistance. One can then deploy gene editing of the target gene(s) or pathway(s) within susceptible cultivars to facilitate host resistance. Recent studies have indicated that when attacked by root parasitic plants, such as *Striga* or *Orobanche*, host plant cells can trigger the immune response, resembling gene-for-gene interactions between hosts and pathogens such as microbes or insects (Fishman and Shirasu, 2021; Jhu and Sinha, 2022). Initially, pathogen-triggered immunity (PTI) is induced upon *Striga* detection, which activates mechanical and biochemical defences in host plant cells. However, *Striga* suppresses PTI and promotes parasitism by injecting effector-like molecules into host plant cells (Li and Timko, 2009). If the host is resistant, effector-triggered immunity (ETI) is activated, leading to programmed cell death, preventing further parasitic growth.

Previously, diverse modes of post-attachment resistance—including mechanical-based resistance and a likely HR—were found in a variety of sorghum cultivars (Kavuluko *et al.*, 2021). Collectively, these show a mechanical barrier-type resistance where increased cell wall thickness could prevent *Striga* ingress, a HR with localized cell necrosis at the host–parasite junction, or deposition of secondary metabolites such as polyphenols. Since these represent a typical onset of an immune response, it is likely that these mechanisms can be attributed to PTI, ETI, or both.

Mutinda *et al.* investigated five sorghum genotypes with documented *Striga* post-attachment resistance phenotypes (Kavuluko *et al.*, 2021) and revealed genotype-specific gene expression signatures underlying two major resistance mechanisms. Biological processes enriched within these molecular signatures were used to classify responses to *Striga* into pattern-triggered immunity (PTI) and effector-triggered immunity (ETI) in these five cultivars (Mutinda *et al.*, 2023).

To date, it was unclear whether a sorghum genotype could possess PTI and ETI simultaneously. Mutinda *et al.* discovered that downstream transcriptional responses of PTI and ETI could co-exist within a single genotype, although some genotypes probably rely predominantly on only one mode of defence. This discovery explains previously observed differences in the efficiency of inducing HR and changes in cell wall composition (Kavuluko *et al.*, 2021). HRs were observed at 90% of the host–parasite contact sites within the root system, while the formation of a cell wall-based barrier was observed in only 50% of *Striga* attachment sites of a given root system, depending on the sorghum genotype. Therefore, certain resistant sorghum genotypes may have evolved the ability to deploy multiple forms of resistance, enhancing their defence mechanisms and increasing their resilience against root-parasitic plants.

Mutinda *et al.* further compiled candidate gene lists for PTI and ETI, which revealed the up-regulation of several sorghum genes whose homologues are associated with HRs and systemic acquired resistance (SAR) pathways in other species, including a pathogenesis-related thaumatin-like gene. They hypothesized that sorghum recognizes specific molecules released during *Striga* infection, such as damage-associated pathogen patterns (DAMPs), and then triggers downstream HRs and mechanical barrier resistance.

### Hypersensitive responses (HRs)

HRs are a well-known defence mechanism in plants, characterized by localized cell death and necrosis at the site of pathogen infection. The observations of localized necrosis upon *Striga* infection in sorghum cultivars IS14963 and IS41724 support an HR. Similar HR mechanisms have been observed in the *Striga* resistance responses of several other host species where haustorium penetration is disrupted (Fig. 1C) (Li and Timko, 2009; Fishman and Shirasu, 2021; Jhu and Sinha, 2022). For example, the rice cultivar Nipponbare, which exhibits strong post-attachment resistance, up-regulates a HR-induced protein upon *Striga* infection (Swarbrick *et al.*, 2008). Similarly, one resistant cowpea cultivar detects unidentified signals from *Striga gesnerioides*, potentially pathogen-associated molecular patterns (PAMPs) or DAMPs, or avirulence (Avr) proteins, triggering activation of a positive regulator of HR (Li and Timko, 2009). This activation leads to localized necrosis upon infestation by *S. gesnerioides*, providing post-attachment resistance in cowpea. However, a specific race of *S. gesnerioides* can overcome this defence response by secreting a small effector that inhibits the positive regulator of HR, resulting in rendering the resistant host cultivar susceptible (Li and Timko, 2009). Critical to any HR is a host gene which has co-evolved with a respective pathogen to detect a pathogen effector via a specific receptor. This interaction initiates signal transduction cascades, culminating in an HR (Fig. 1C). The target receptors in these sorghum cultivars remain to be determined, although there was evidence of several candidate nucleotide-binding site leucine-rich repeat (NBS-LRR) genes up-regulated in IS9830 and N13.

### Cell wall modifications

Many resistant host species modify their cell wall composition to enhance this physical barrier against *Striga*, often through lignin deposition. Mutinda *et al.* emphasized the significance of the lignin biosynthesis pathway in *Striga* resistance, observing that genotypes with cell wall-based resistance showed up-regulation of essential lignin biosynthesis genes (Fig. 1D). This finding is consistent with previous studies conducted on *Striga*-infected cowpea and rice (Huang *et al.*, 2012; Mutuku *et al.*, 2019), further highlighting the role of lignin biosynthesis in conferring resistance (Yoshida and Shirasu, 2009).

In addition to lignification, callose deposition is another defence mechanism employed by plants that strengthens the plant cell wall to resist pathogen infections (Bacete *et al.*, 2018). Mutinda *et al.* identified a gene called *Glucan Synthase-Like 10* (*GSL10*), which catalyses callose production. This discovery aligns with previous studies highlighting the importance of callose deposition in resisting attacks from other parasitic plant–host systems (Fig. 1D), although callose remains to be observed within the sorghum cultivars characterized.

## Future research and perspectives

Overall, Mutinda *et al.* shed light on the transcriptional basis of observed sorghum resistance mechanisms against *Striga* and identified candidate genes for further enhancing sorghum through breeding or genetic modifications. Future research should focus on localized, inducible modes of resistance. The transcriptome changes should be assessed specifically at the attachment sites, comparing responses at successful and blocked attachment sites. Finally, it is vital to acknowledge the practical challenges of implementing host resistant varieties and promote integrated management approaches. We propose future research directions for integrated crop management to enhance crop improvement.

### Inducible defence responses

Inducible defence responses activated upon pathogen detection allow a plant to allocate resources efficiently and to enhance survival and reproductive success (Shudo and Iwasa, 2001). Known post-attachment resistances types are mainly inducible mechanisms triggered by the presence of parasitic plants (Fishman and Shirasu, 2021; Jhu and Sinha, 2022; Jhu *et al.*, 2022). This interaction between the host and parasitic plant might direct the co-evolution of resistance mechanisms and lead to different sorghum genotypes domesticated in different regions of Africa exhibiting various gene expression profiles and resistance responses (Kavuluko *et al.*, 2021; Mutinda *et al.*, 2023).

Constitutive defence mechanisms could hamper crop growth. For example, knocking out a negative regulator in resistant tomato cultivars leads to constitutive lignin accumulation, increasing resistance to *Cuscuta* but stunting vegetative growth (Jhu *et al.*, 2022). Thus, genetic engineering and breeding towards *Striga* resistance should focus on inducible defence responses (Gurr and Rushton, 2005).

### Cell type- or tissue-specific defences

**Fig. 2.**
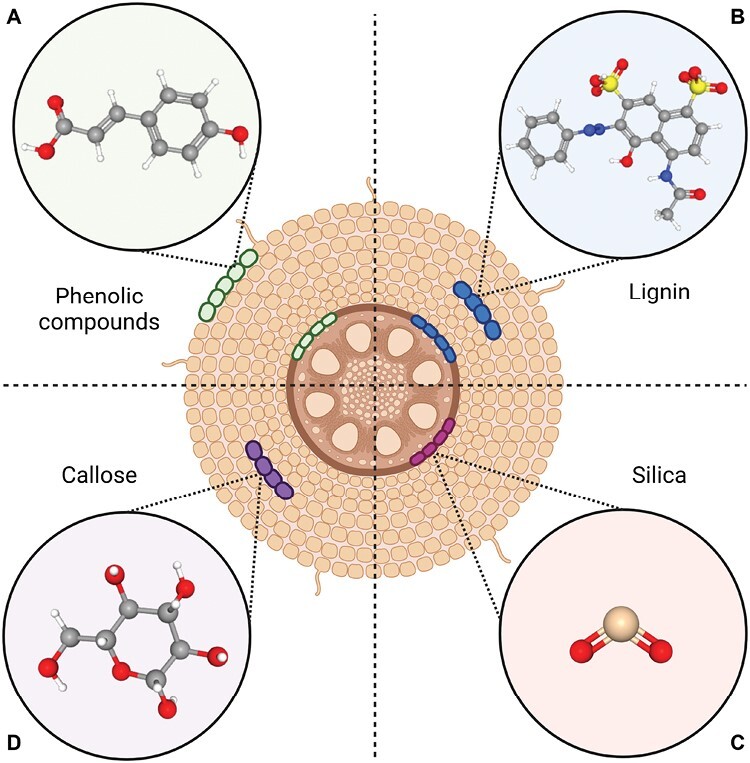
Cell type-specific barriers and defence mechanisms safeguarding host plants against root-parasitic plant invasion. Plant cell type-specific barriers and defence mechanisms play a vital role in protecting plants against root-parasitic plant invasion. Notable examples of these protective barriers include: (A) concentrated accumulation of phenolic compounds in the epidermis and endodermis, (B) localized deposition of lignin in the cortex and endodermis, (C) targeted accumulation of silica in the endodermis, and (D) confined build up of callose in the cortex. Three-dimensional structure images of phenolic compounds (4-hydroxycinnamic-acid, Compound CID: 637542), lignin (Compound CID: 175586), silica (silicon dioxide, Compound CID: 24261) and callose (beta-d-glucose, Compound CID: 64689) are exported from PubChem. This figure is created with https://www.biorender.com/.

Cell type-specific barriers and defence mechanisms are crucial for plants to resist root-parasitic plant invasion (Hu *et al.*, 2020; Kawa and Brady, 2022, preprint). Epidermal phenolic compounds physically redirect or impede parasitic plant growth, while lignin or callose barriers in the cortex hinder parasite progression (Fig. 2) (Yoshida and Shirasu, 2009; Yoder and Scholes, 2010). Increased accumulation of lignin or other phenolic compounds and silica deposition in the endodermis prevent the parasitic plant from reaching the vasculature, while reinforcing xylem vessels with additional lignin restricts establishment of xylem–xylem connections (Fig. 2) (Yoshida and Shirasu, 2009; Mutuku *et al.*, 2019). These cell type-specific defence mechanisms play a crucial role in preventing the invasion and establishment of parasitic plants. Localized defence mechanisms at the parasite–root interface minimize energy costs and unintended effects. New technologies such as single-cell RNA-sequencing or spatial transcriptomics could yield more comprehensive information on the spatiotemporal patterns of regulation of the post-attachment resistance. Incorporating cell-specific defences into *Striga* resistance breeding programmes could enable the development of crop cultivars with enhanced protection.

### Integrated management of *Striga*

To effectively control *Striga*, an integrated approach is recommended, combining cultural practices, chemical control, breeding solutions, and bioinoculants. Strategies such as intercropping, water management, crop rotation, trap crops, and fertilization have been employed to manage *Striga* (David *et al.*, 2022). Chemical compounds that mimic strigolactone action, known as suicidal germination agents, offer promise in allowing *Striga* to sprout without a host before crop planting (Kountche *et al.*, 2019). Fungal isolates pathogenic to *Striga* have been successfully used in maize fields to reduce *Striga* infection (Nzioki *et al.*, 2016). Bacterial species degrading haustorium-inducing factors or inducing mechanical barriers in sorghum roots have been isolated (Kawa *et al.*, 2022, Preprint). Additionally, using beneficial microorganisms as bioinoculants can promote plant health and inhibit *Striga* growth and attachment (Jamil *et al.*, 2021; Abdullahi *et al.*, 2022).

Combining two or more methods has shown significant results. For instance, combining host resistance with a beneficial fungus that produces myco-herbicides has considerably reduced *Striga* (Bàrberi, 2019). The combination of phosphate fertilizer, a resistant host variety, and rhizobium inoculation reduced *Striga* and increased the grain yield of cowpea (Abdullahi *et al.*, 2022; David *et al.*, 2022). Intercropping maize cultivars that are *Striga* resistant and herbicide resistant with legume crops has suppressed *Striga* seed germination and reduced the *Striga* seed bank in the soil (Kanampiu *et al.*, 2018). Utilizing host plant varieties with pre-attachment resistance could induce suicidal germination in *Striga* and therefore be an effective approach for reduction of *Striga* seed banks (Kountche *et al.*, 2019). Furthermore, when combined with germination stimulants, cereal–legume crop rotation can significantly reduce *Striga* seeds in the soil (Jamil *et al.*, 2021). These results demonstrate that to achieve effective and sustainable *Striga* control, an integrated approach is recommended (Mwangangi *et al.*, 2021; Abdullahi *et al.*, 2022).
